# Discriminating signal from noise in the fossil record of early vertebrates reveals cryptic evolutionary history

**DOI:** 10.1098/rspb.2014.2245

**Published:** 2015-02-07

**Authors:** Robert S. Sansom, Emma Randle, Philip C. J. Donoghue

**Affiliations:** 1 Faculty of Life Sciences, University of Manchester, Manchester M13 9PT, UK; 2 School of Earth Sciences, University of Bristol, Life Sciences Building, 24 Tyndall Avenue, Bristol BS8 1TQ, UK

**Keywords:** eustasy, competitive replacement, facies bias, ostracoderm, gnathostomes, diversity

## Abstract

The fossil record of early vertebrates has been influential in elucidating the evolutionary assembly of the gnathostome bodyplan. Understanding of the timing and tempo of vertebrate innovations remains, however, mired in a literal reading of the fossil record. Early jawless vertebrates (ostracoderms) exhibit restriction to shallow-water environments. The distribution of their stratigraphic occurrences therefore reflects not only flux in diversity, but also secular variation in facies representation of the rock record. Using stratigraphic, phylogenetic and palaeoenvironmental data, we assessed the veracity of the fossil records of the jawless relatives of jawed vertebrates (Osteostraci, Galeaspida, Thelodonti, Heterostraci). Non-random models of fossil recovery potential using Palaeozoic sea-level changes were used to calculate confidence intervals of clade origins. These intervals extend the timescale for possible origins into the Upper Ordovician; these estimates ameliorate the long ghost lineages inferred for Osteostraci, Galeaspida and Heterostraci, given their known stratigraphic occurrences and stem–gnathostome phylogeny. Diversity changes through the Silurian and Devonian were found to lie within the expected limits predicted from estimates of fossil record quality indicating that it is geological, rather than biological factors, that are responsible for shifts in diversity. Environmental restriction also appears to belie ostracoderm extinction and demise rather than competition with jawed vertebrates.

## 1. Introduction

The diversity and disparity of living vertebrates are dominated by the gnathostomes (jawed vertebrates), with jawless vertebrates comprising just two conservative lineages, the hagfishes and lampreys. However, jawed vertebrates only assumed dominance in the Devonian (419–359 Ma); for the preceding 100 million years (Myr), vertebrate communities were dominated by the ‘ostracoderms’, an entirely extinct grade of jawless fishes characterized by an extensive armour-like dermal skeleton. The ostracoderms are arrayed phylogenetically in a series of successive sister clades to the jawed vertebrates, evidencing the gradual assembly of the gnathostome bodyplan [1,2]. Hence, there has been considerable interest in what the fossil record reveals of this formative episode of vertebrate evolutionary history.

To date, the evolutionary dynamics of events surrounding the emergence of jawed vertebrates have been read literally from the stratigraphic record [3,4]. Examples include the hypotheses that the vertebrate skeleton evolved in response to predation by eurypterids [5], and that ostracoderms were competitively displaced by jawed vertebrates [6,7], both of which are based on coincident patterns of raw diversity. Similarly, flux in ostracoderm diversity has been rationalized as shifts in the rate of diversification associated with changes in ecology and competition for habitats [6–11]. However, it is now widely acknowledged that the fossil record is biased by secular variation in the rock record, such that it has become appropriate to assume a null model that stratigraphic variation in rock area explains sampled diversity [12,13]. There is already some evidence to suspect that sampled diversity of ostracoderms is biased by the nature of the rock record. For instance, plesiomorphic and derived representatives of the clades have coincident first stratigraphic records, betraying a cryptic prehistory [3,4,9,14]. Any such pre-fossil history necessitates a dramatic revision of contemporary scenarios that seek to explain the evolutionary origin of gnathostomes, because ostracoderms have the most relevance to our understanding of the gnathostome stem and the long ghost ranges subtended from it. Furthermore, the component ostracoderm clades exhibit strong facies associations and, consequently, shifts in diversity appear to coincide with changes in facies and sea-level [3,4,14–16]. By framing the analyses of biodiversity in the light of palaeoenvironment and potential geological biases, we aim to assess the veracity of the fossil record of this most formative episode in vertebrate evolutionary history.

## 2. Material and methods

### (a) Biodiversity

To test the hypothesis that ostracoderm diversity is explained by secular bias in facies representation in the rock record, we determined the genus-level diversity of representative ostracoderm clades and the number of fossiliferous formations in which these ostracoderm groups are encountered. Data were collected for the four main clades of ostracoderms—osteostracans, galaeaspids, thelodonts and heterostracans, each being major plesions on the gnathostomes‘ stem-lineage [15,17–19]. Palaeobiology database records are incomplete for these clades [20]. Instead, a new dataset was compiled based on an exhaustive literature search for the four clades (principally Sansom [21] and P. Janvier (2004, unpublished data) for osteostracans, Zhu & Gai [22] for galeaspids and Märss *et al*. [23] for thelodonts, and a new data synthesis for heterostracans). As such, the new dataset includes all published occurrences of ostracoderms from these clades and, thus, the vast majority of ostracoderms (very few genera of Anaspida exist, and they are restricted in stratigraphic distribution). Genus-level phylogenies are available for Osteostraci [21], Galeaspida [22] and Thelodonti [24], which enable the inclusion of ghost ranges. For the Osteostraci, the age and palaeoenvironment of each osteostracan-bearing locality was reviewed (electronic supplementary material, table S1). Monophyly of each of the four ostracoderm clades is assumed here [20,25]. However, to control for the possibility that they are paraphyletic grades, major subclades of thelodonts and osteostracans are subjected to separate analyses. Genus-level diversity for jawed vertebrates was obtained from Sepkoski's compendium [26]; this is limited in some regards [20] and, as such, the jawed vertebrates’ diversity curve is used for comparative purposes only.

### (b) Geological biases

To determine whether flux in diversity can be accounted for by variation in the availability of appropriate rock sequences, we used in proxy the number of fossiliferous horizons or formations from each interval [27–29]. Horizons from close geographical locations are not distinguished (electronic supplementary material, table S1). Plotting the number of osteostracan-, galeaspid-, thelodont- or heterostracan-bearing rock formations against the number of genera from each interval reveals the relationship between fossil availability and diversity per unit time. In some instances, it is necessary to use first differences in order to eliminate the role of autocorrelation in time-series data [30–32].

To determine the degree to which palaeontological sampling reflects the true stratigraphic range of taxa, we calculated confidence intervals on their first appearance. Assuming that fossils are randomly distributed and have a constant recovery potential, 95% and 99% confidence intervals, respectively, for the timing of origination of a clade were calculated using Marshall's formula [33]:2.1
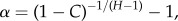
where *α* is the fraction of the known stratigraphic range of the clade, *C* is the confidence interval and *H* is the number of known fossiliferous horizons for the clade.

This model assumes, however, that fossils are randomly distributed and have a constant recovery potential; this assumption is not met in the fossil record of ostracoderms [3] nor the fossil record more generally [34]. To assess the influence of abiotic factors on fossil recovery potential, sea-level and total rock-outcrop area were plotted against the number of fossiliferous horizons from each geological time interval for each clade. An emphasis is placed on local rather than global patterns as it has already been observed that biases can be highly regional [14] ([35,36] for Devonian of Euramerica; [37] for Silurian of China; [38,39] for Silurian of Euramerica; [40] for Ordovician of Laurentia and Yangtze platform; [12] for total rock-outcrop area in Western Europe). In instances where the recovery potential of a clade correlates with an abiotic factor, the relationship between that abiotic factor and the number of fossiliferous horizons can be used to calculate more realistic confidence intervals using Marshall's formula for calculating confidence intervals when fossil recovery potential is non-random [41]:2.2
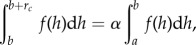
where *b* is the lowerbound of the stratigraphic range of a clade, *a* the upperbound, *r*_c_ the length of the confidence interval and *f*(*h*)d*h*, the function of recovery potential with respect to time. This formula uses the same rationale as that used for uniform recovery, but uses an abiotic factor as a proxy for recovery potential. In this case, 95% and 99% confidence intervals of the origination dates of clades are forecast using a model of recovery potential based on the quantitative relationship identified between number of fossil-bearing formations and an abiotic factor.

## 3. Results

### (a) Biodiversity

**Figure 1.**
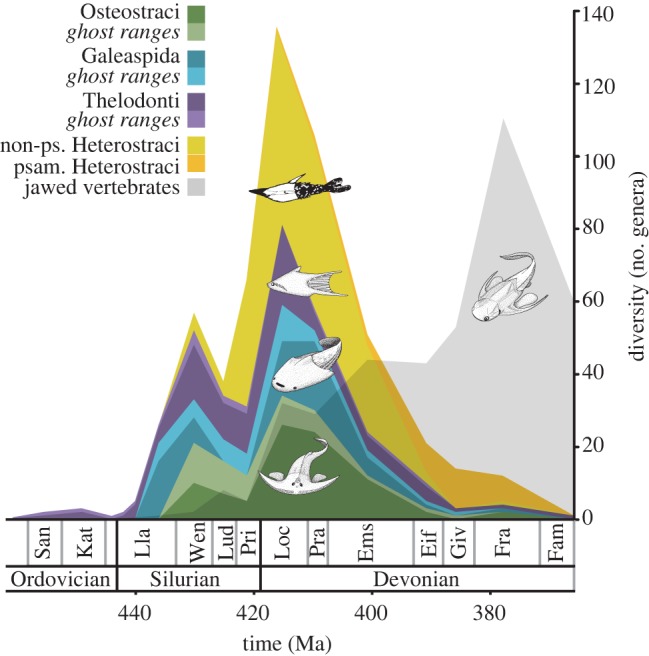
Diversity of ostracoderms through time. Stacked numbers of genera at each interval is shown for osteostracans (green), galeaspids (blue), thelodonts (purple) and heterostracans (non-psammosteid, yellow and psammosteid, orange). Additional ghost ranges in lighter shades for osteostracans, galeaspids and thelodonts. Diversity of jawed vertebrates [26] are overlain in grey. Absolute ages and standard stages (Sandbian through to Famennian) from [42].

The reconstructed diversity curves for ostracoderms (figure 1) follow a similar pattern in each group; there was an early peak in diversity in the Silurian followed by a drop in the Upper Silurian and then larger maximum peak in at the Early Devonian (Lochkovian) followed by gradual decline towards the end of the Devonian. This matches the periods of ‘origination, survival, radiation and decline’ described for Chinese vertebrates [7]. The principal deviations from this pattern are the earlier initial origin of thelodonts in the Ordovician, the slightly earlier rise in diversity of galeaspids in the Silurian of China, a relative paucity of Silurian heterostracans and the decline in diversity of thelodonts in the Early Devonian. Another principal source of deviation from the general pattern is the Middle–Late Devonian record of heterostracans. Unlike the other ostracoderms clades, the rate of their Devonian decline is slower. This is attributable, almost entirely, to the diversification of the Psammosteidae. The Ludlow and Pridoli record of South China is missing owing to uplift of the Cathaysian upland [37,43] and is treated, therefore, as a gap. Despite the osteostracan and galeaspid clades occurring on two geographically disparate palaeocontinents, their diversity curves (including ghost ranges) follow a near-identical pattern during the Devonian. Jawed vertebrates [26] show a very different pattern from ostracoderms: low diversity in the Silurian and gradual increase in the Devonian, achieving a maximum in the Upper Devonian (Frasnian). More complete records for jawed vertebrates might diverge from this particular curve [20], but we anticipate that the general pattern (i.e. initial Silurian radiation and increasing diversity in Devonian) is robust to sampling.

### (b) Geological biases

**Figure 2.**
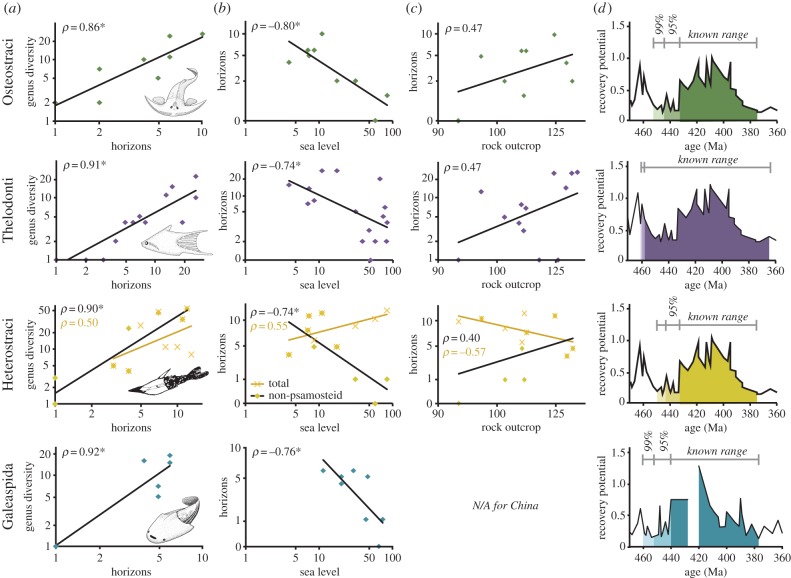
Quantitative assessment of the diversity and recovery potential of the Osteostraci, Thelodonti, Heterostraci (non-psammosteid, yellow/black, and total, orange/brown) and Galeaspida. (*a*) The positive relationships between diversity (number of genera, without ghost ranges) and record quality (number of horizons); (*b*) Inverse relationships between the number of horizons per interval and average sea-level per interval (percent maximum); (*c*) Non-significant correlations between the number of fossiliferous horizons and total rock outcrop area (number of maps of Western Europe from [12]); (*d*) Fossil recovery potentials through time for Osteostraci, Thelodonti, non-psammosteid Heterostraci and Galeaspisa based upon the inverse logarithmic relationship calculated in (*b*). The dark area represents the known ranges of the clades (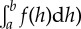), whilst the lighter (95%) and lightest areas (99%) under the lines represent the confidence intervals calculated as a proportion of the area under the line for the known range 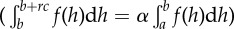. All axes, except time, have been logged. Significant correlations marked with asterisk.

Osteostracans, galeaspids and thelodonts show a strong correlation between genus diversity and number of fossil-bearing horizons (figure 2*a*; Pearson's correlation coefficients (*ρ*) of 0.86, 0.92 and 0.91, respectively, all *p* < 0.01). This is not the case for heterostracans (*ρ* = 0.50, *p* > 0.10); the Middle–Late Devonian Psammosteidae have a very different relationship between horizons and genera from the rest of the heterostracans as well as that of other ostroacoderms. Non-psammosteid heterostracans do exhibit a significant positive correlation (*ρ* = 0.90, *p* < 0.01). Using the identified relationships between the number of fossiliferous horizons and the number of genera for each group, only Telychian thelodonts have a residual value greater than two standard deviations in magnitude (see data in the electronic supplementary material). As such, the vast majority of diversity changes in these groups are within the range expected given the number of fossiliferous horizons, a proxy for the extent of rock available for sampling per unit time [29,30]. These patterns remained unchanged after detrending data for autocorrelation using first differences [30,31]. The smaller sample size and the evident gap in their fossil record made detrending the galeaspid data impractical.

Occurrence data indicate that the Osteostraci are restricted to the shallowest near-shore marine/marginal marine and freshwater environments only (electronic supplementary material, figure S1 and table S1). Similar interpretations have been made of Galeaspida [15,44]. Indeed, the initial appearance of the osteostracan and galeaspid lineages is coincident in all localities with a drop in sea level from deep marine to shallow marine/marginal marine facies ([14]; electronic supplementary material, figure S2). As such, it is anticipated that sea-level-driven shifts in sedimentary facies dictate the stratigraphic occurrence of ostracoderms. This relationship is confirmed by the strong relationship identified between the secular variation of sea-level and the number of fossil-bearing horizons (figure 2*b*) for osteostracans (*ρ* = −0.80, *p* = 0.01), galeaspids (*ρ* = −0.76, *p* = 0.03), thelodonts (*ρ* = −0.74, *p* = 0.001) and non-psammosteid heterostracans (*ρ* = −0.74, *p* = 0.02). The recovery potential of ostracoderm fossils is therefore proportional to the inverse of sea-level. The same significant relationship is observed in detrended data for osteostracans (*ρ* = −0.76, *p* = 0.02) and non-psammosteid heterostracans (*ρ* = −0.68, *p* = 0.04), but not for thelodonts (*ρ* = 0.29, *p* = 0.28). Comparable analyses for dinosaurs find significant relationships between sea-level and diversity, but as a result of autocorrelation only [31]. A more direct measure of the fossil record, rock outcrop area, is found to be uncorrelated with the number of horizons for osteostracans, thelodonts and heterostracans (figure 2*c*, *ρ* = 0.47, 0.47, 0.40, respectively, all *p* > 0.10). Total rock-outcrop of Western Europe is an unsuitable proxy for the influence of abiotic factors on fossil recovery potential of galeaspids as this clade is restricted palaeogeographically to the regions that comprise modern SE Asia.

**Figure 3.**
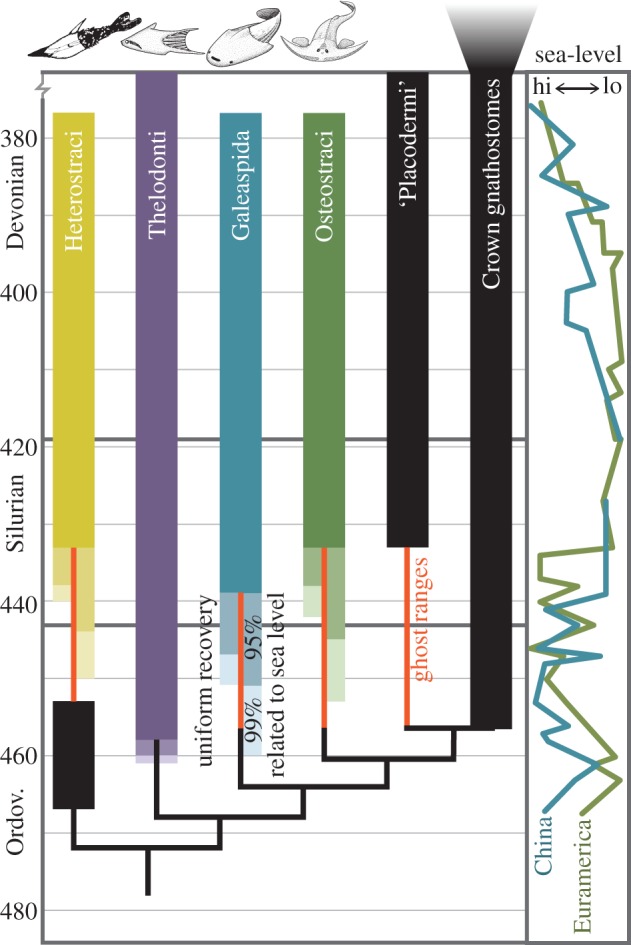
Stratigraphic ranges of stem- and crown-gnathostome clades in the Middle Palaeozoic. Thin black lines represent the phylogenetic branching, with ghost ranges highlighted in orange. Confidence intervals (95% bars in lighter shades, 99% bars in lightest shades) were calculated on the basis of random distribution of fossil horizons (uniform recovery, left bar) or using the relationship between sea-level curves (graph, right) and number of horizons to forecast recovery potential (figure 2*d*; right bar). Confidence intervals relating to sea level account for long ghost ranges of Osteostraci and Galeaspida, and the gap between Heterostraci and non-heterostracan pteraspidimorphs.

The strict origination dates using known stratigraphic ranges are 433 million years ago (Ma) for osteostracans and heterostracans, 439 Ma for galeaspids and 458 Ma for thelodonts (figure 3, Wenlock, Llandovery and Sandbian, respectively). Using the total number of horizons (H), the known stratigraphic range, and the formulae above, 95% and 99% confidence intervals for the date of origination were calculated for each ostracoderm group. Using models of constant recovery potential, 95% confidence intervals range from 2 to 8 Myr (figure 3 and electronic supplementary material, data). In order to take into account non-constant models of recovery for ostracoderms, the significant quantitative relationship between sea-level and number of horizons for each group was used to reconstruct fossil recovery potential curves from Middle Ordovician to the Upper Devonian (figure 2*d*). These, together with the proportion of the known ranges (*α* from formula 1), were used to calculate 95% and 99% confidence intervals for the origination date of each clade (figure 3; formula 2; electronic supplementary material, data). For the osteostracans, galeaspids and non-psammosteid heterostracans, sea-level-based confidence intervals indicate potential origins much earlier than models of constant fossil recovery potential (20, 21 and 17 Myr before first-known occurrence, respectively, at 99%, compared with 9, 12 and 4 Myr). Confidence intervals for the origin of thelodonts are the same whether using models of uniform recovery or non-random eustasy models (both 2 Myr).

Splitting ostracoderm clades (plesions) into subclades (paraplesions) serves only to increase confidence intervals. The two major divisions of osteostracans (eucornuates and thyestiids) [21] have fewer horizons than the total group (20 and 13, respectively, compared with 36 total osteostracan) and, thus, have higher 95% confidences intervals for origination (8 and 9 Myr compared with 5 Myr for total group). Using the sea-level recovery potential relationship for Osteostraci provides longer confidence intervals still (28 Myr for Thyestiida, compared with 20 Myr for Osteostraci). A similar pattern is seen for the two major divisions of thelodonts (Furcacaudiformes and allies versus Shieliiformes, Phlebolepidiformes and allies [23]), where each has longer 95% confidence intervals than the combined clade (6.5 and 3.5 Myr, for the respective paraplesions).

## 4. Discussion

### (a) Originations

The first-known occurrences of the Osteostraci, Heterostraci and Galeaspida (Wenlock, Wenlock and upper Llandovery, respectively) are appreciably younger than those of closely related clades (thelodonts, jawed vertebrates and arandaspids) which all originate in the Ordovician [10,11,16,44]. Because sister lineages are by definition, of equal age, the stratigraphic ranges of these clades appear inconsistent with current understanding of their phylogenetic relationships (figure 3). The occurrence of jawed vertebrates in the Ordovician necessitates long ghost ranges for the Osteostraci and Galeaspida. This is true whether they are interpreted as crown-gnathostomes (e.g. acanthodian/chondrichthyan), or as stem–gnathostomes [11,16,20]. In any case, confidence intervals on the age of origin of these lineages indicate that the fossil records of ostracoderms are a poor approximation of their antiquity. Using models of constant recovery potential at 95% and 99% confidence, the envelope of possible origination dates extends deep into the earliest Silurian for Osteostraci and Heterostraci, and to the Upper Ordovician for Galeaspida (figure 3 and electronic supplementary material, data); the confidence interval on the first occurrence of Thelodonti is much shorter and extends their potential range only marginally (figure 3). Investigations of the geological biases indicate that the fossil record of ostracoderms is tied strongly to sea-level change. Models of recovery potential that reflect more realistic geological biases (i.e. sea-level changes), extend the potential origins of Osteostraci and Galeaspida deeper into the Upper Ordovician ‘Talimaa's gap’ [11,15], a facies-shift associated with the end-Ordovician glaciation events. Non-preservation of these clades during the Early Silurian likely reflects a global high-stand at sea-level. Heterostraci (excluding Psammosteidae) show the same pattern (see below). Conversely, the fossil record of thelodonts is interpreted as less ecologically restricted [15,22,45,46] and it is evidently less impacted by sea-level-driven facies change (figure 2*b*). Confidence estimates on origination dates for Thelodonti, using either constant recovery potential or models taking sea-level changes into account, are only marginally older than known first occurrences (figure 3). In all instances, the compiled nature of the sea-level curve is an imprecise estimate, but the eustatic pattern of Llandovery high stands and Late Silurian/Early Devonian low stands yield similar confidence intervals. Similar facies biases might also account for the relatively late stratigraphic appearance of placoderms (figure 3). Interpreting the record of ‘Placodermi’ is, however, complicated by their likely paraphyly and more varied ecology [34,47–49]; component placoderm plesions will need to be evaluated individually in future studies. If ostracoderm clades are treated as paraphyletic plesions, their confidence intervals on origination dates are even longer; analysis of osteostracan and thelodont subclades (above) reduces the number of horizons, exacerbating perceptions of fossil record incompleteness.

Our confidence intervals on the originations of Osteostraci and Galeaspida are far more consistent with stem–gnathostome phylogeny and, as such, stratigraphic ranges do not give us reason to doubt reconstructions of stem–gnathostome relationships. However, there is no known record of the early stages in the evolutionary history of these groups, raising serious concerns over received knowledge of the sequence of character evolution in the gnathostome stem. While palaeoenvironments appropriate for the fossilization of Osteostraci and Galeaspida are not known and possibly not preserved for the periods that are critical to our understanding of their early evolution (Upper Ordovician and lower Llandovery), a micropalaeontological approach, combined with more disparate palaeobiogeographic sampling, might provide insights into this otherwise cryptic interval of vertebrate evolutionary history [10,15,50,51]. However, few reliable anatomical characters are available to evidence the phylogenetic affinity of the Ordovician microremains [24].

### (b) Diversifications

The Early Devonian peaks in diversity appear to be coincident with periods of maximum morphological disparity, faunal turnover and environmental innovation, potentially betraying a real biological signal of diversification. Different clades of osteostracan, galeapid and heterostracan ‘radiate’ simultaneously into similar and disparate morphospaces (e.g. extended cornual and rostral processes), perhaps indicating similar ecological pressures upon the clades and diversification into comparable niches. There also appears to be turnover within ostracoderm clades between the Silurian and the Devonian, with few genera common to both periods (i.e. non-corunates and thyestiid osteostracans are replaced by Benneviaspida and Zenaspida, whereas early diverging galeaspids and Eugaleaspidiformes are replaced by Polybranchiaspidida). Furthermore, the Early Devonian peak in osteostracan biodiversity is correlated with an important palaeoenvironmental change—the transition from marine to fresh water. At least two marine-to-freshwater transitions occur within Osteostraci in the early Devonian: one in eucornuates, another in Kiaeraspidoidae and potentially *Ilemoraspis* (electronic supplementary material, figure S1; [52]). However, it would be unwise to consider the Early Devonian radiation as an episode of rapid diversification in response to a key innovation (euryhalinity, i.e. tolerance of a wide range of salinities) and increase of available ecospace, because the same Early Devonian peak in diversity occurs in the entirely marine Galeaspida. Without a phylogenetic framework for Heterostraci, it is not appropriate to evaluate turnover in the same way.

The Early Devonian ‘radiations’ are also matched with a marked increase in the deposition of shallow-water sediments across both Euramerica (Caledonian orogeny) and South China (increasing shallow–continental shelf area). Furthermore, there is an absence of ecologically appropriate strata preceding the Upper Silurian in South China and Spitsbergen, which may account for the Silurian ghost ranges of clades from these particular regions. The apparently sudden ‘burst’ in ostracoderm diversity at the beginning of the Devonian as well as the subtle differences in origination dates of the Osteostraci and Galeaspida may therefore be an artefact of local facies changes rather than biological response ([8,9,34]; electronic supplementary material, figure S2). The fauna of the Upper Silurian is either depauperate or absent, but it clearly survived this interval given its occurrence in the preceding Lower Silurian and subsequent Early Devonian strata. Furthermore, models of recovery potential estimate appreciably earlier originations than current records suggest. As such, our analyses of stratigraphic biases highlight missing records of ostracoderms and undermine the interpretation of raw diversity shifts as biological responses because suitable ostracoderm environments must have existed, but they were not preserved. Whether biological response to changing availability of ecospace [7] or geological bias caused by variable preservation [3,8,14,34], it is clear that the Siluro-Devonian fossil record of ostracoderms is strongly tied to facies changes.

### (c) Extinctions

New data from the ostracoderms can be used to shed light on the traditional hypothesis that jawless vertebrates were replaced by jawed vertebrates in an episode of competitive replacement towards the end of the Devonian. Ostracoderms and jawed vertebrates fit many of the criteria necessary for competitive replacement: the ostracoderm decline in diversity during the Middle Devonian and eventual extinction in the Late Devonian are contemporaneous with the taxonomic ascendency of jawed vertebrates, thus matching the ‘double-wedge’ pattern that would indicate competitive replacement (figure 1 [26,53,54]). Ostracoderms and jawed vertebrates have overlapping stratigraphic, geographic and body size ranges, but it is less clear whether they occupy similar ecological ranges. Where data are available, ostracoderms are interpreted as deposit feeders or microphagous suspension feeders [25,55–57], which differs clearly from the far more diverse and predatory diets of placoderms and crown-gnathostomes [15,22,58–61]. Furthermore, osteostracans, galeaspids and to a lesser extent thelodonts and heterostracans are restricted to shallow water or continental palaeoenvironments (figure 2 and electornic supplementary material, table S1), whereas jawed vertebrates are less restricted, being found in deeper and more varied habitats. Furthermore, the benthic mode of life of osteostracans and galeaspids implied by trace fossils [62] and body shape differs from early-jawed vertebrates that (with notable exceptions) are interpreted as active swimmers invading the pelagic realm [49]. One clade of ostracoderms that bucks these trends is the psammosteid heterostracans; unlike all others, they radiate in the Middle/Late Devonian and do not show an inverse correlation with Laurentia sea-level changes. This could reflect regional differences in facies change or ecological differences (psammosteids possess dorsal mouths and ventral keels, unlike other heterostraci or ostracoderms).

In sum, historic patterns of taxonomic diversity and overlapping geographic and stratigraphic ranges are consistent with competitive replacement of jawless vertebrates by jawed vertebrates. Substantial differences in the habitat and diet of these two grades, however, overturn hypotheses of competitive replacement and invite us to explore abiotic factors underlying the demise of ostracoderms [53]. Principal among these is sea-level change. The beginning of the decline of all major clades of ostracoderms is coincident with rising sea-level; this pattern continues for the rest of the Devonian and is within the limits predicted from models based upon sea-level changes.

Thus, the emerging picture is of a loss of diversity of jawless vertebrates, not in response to competition, but in response to a reduction of suitable palaeoenvironments in the Middle and Late Devonian. The limited palaeoenvironments and restricted geodispersal capability [63] of the ostracoderms left them exposed to the drastic geological changes during the Devonian and are likely to have been the key factors in their demise and extinction. Increased dispersal capacity [4,63] and broader ecologies of jawed vertebrates meant that they were better placed to survive and respond to these changing conditions. In order to test such hypotheses, more detailed data are needed on the palaeobiogeography, palaeoecology and phylogeny of individual clades of jawed vertebrates, comparable to that of jawless vertebrates.

## 5. Conclusion

As the closest relatives of jawed vertebrates, the Osteostraci, Galeaspida, Thelodonti and Heterostraci serve as the most suitable groups with which to investigate hypotheses regarding the origins and demise of jawless vertebrates. Data presented here enable elucidation of the patterns and processes regarding the origin, diversification and extinction of the stem–gnathostomes. The restrictive palaeoecology of the ostracoderms resulted in a strong role of facies bias, which pervades our understanding of all of these episodes. On the basis of sea-level changes, phylogenetic inferences and confidence intervals adjusted for non-random sea-level changes, the origins of the Osteostraci, Galeaspida and, potentially Heterostraci, could have occurred appreciably earlier than currently recorded in the fossil record, in the Upper Ordovician. This makes the stratigraphic ranges of these clades more comparable to those of related clades with less restrictive palaeoecologies, which have been less affected by facies bias (i.e. thelodonts and jawed vertebrates) as well as being more consistent with current hypotheses of stem–gnathostome phylogeny. It also highlights important gaps in our knowledge of stem–gnathostomes at an important stage in the evolution of vertebrates and the acquisition of gnathostome characters. Apparent bursts in stem–gnathostome biodiversity at the beginning of the Devonian also occur in response to increased deposition of palaeoenvironments.

Regarding their demise, patterns of diversity of jawless and jawed vertebrates through time may well be consistent with the models of competitive replacement, but differences in diet and habitat make such a process of clade replacement untenable. Instead, data presented here support the idea that the restrictive ecology and limited geodispersal ability of the ostracoderms resulted in them being more adversely affected by changing geological conditions in the Middle and Late Devonian than their jawed relatives.

## Supplementary Material

Supplementary Figures and Table

## Supplementary Material

Supplementary Data
