# Supplementary material for: Discriminating signal from noise in the fossil record of early vertebrates reveals cryptic evolutionary history
Source: Proc Biol Sci. 2015 Feb 7;282(1800):20142245. doi: 10.1098/rspb.2014.2245 (PMC4298210; doi:10.1098/rspb.2014.2245)
Supplement: Supplementary Figures and Table [file rspb20142245supp1.doc]

**ELECTRONIC SUPPLEMENTARY MATERIAL FOR:**

**Discriminating signal from noise in the fossil record of early vertebrates reveals cryptic evolutionary history**

Robert S. Sansom, Emma Randle, Philip C. J. Donoghue

(Link to journal information)


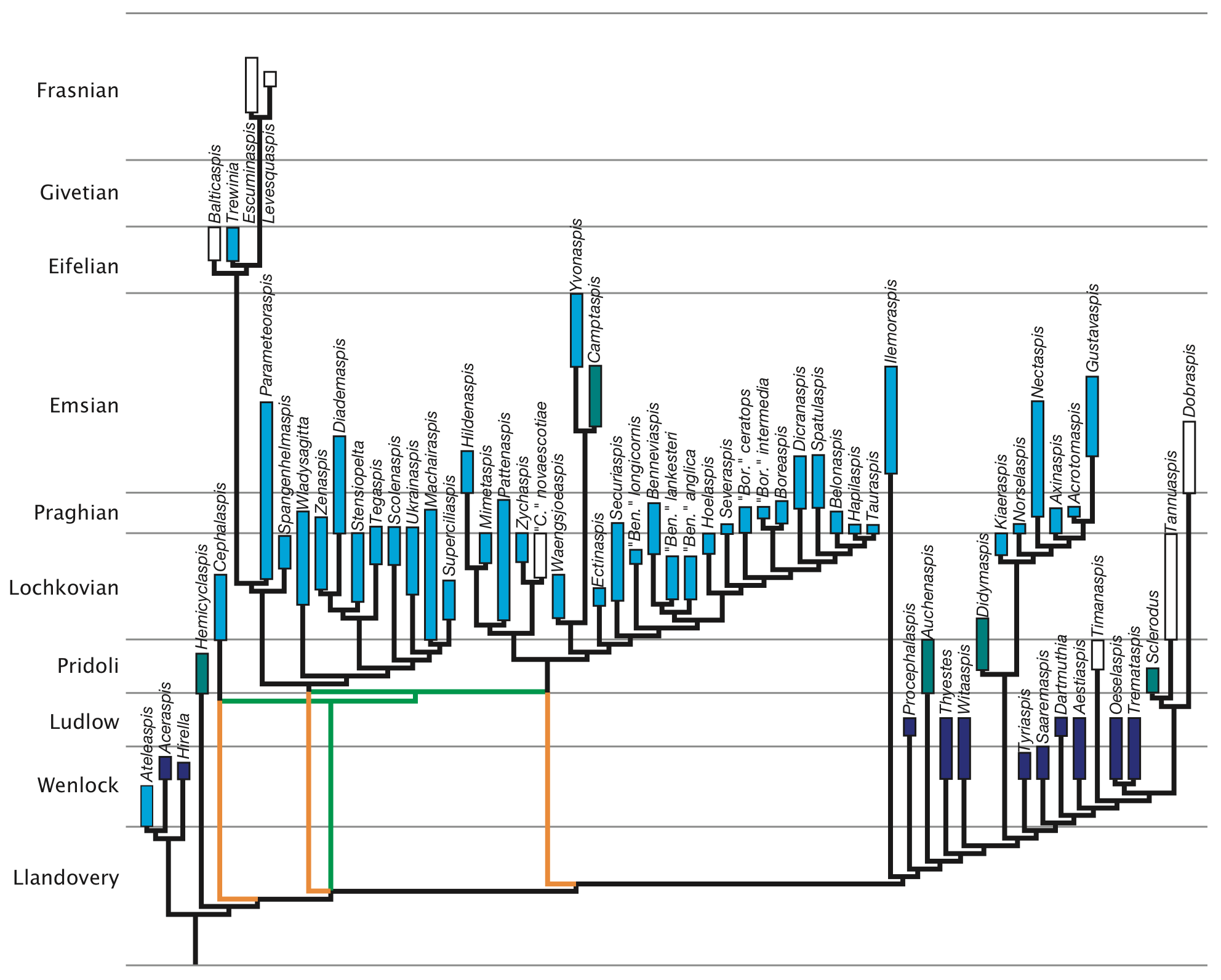
 **Supplementary Figure 1**. Osteostracan phylogeny (Sansom 2009) applied to stratigraphic ranges. The ages and environments are identified from the sources in Supplementary Table 1. Colour of bars represent depositional environment as either marine (dark blue), marginal marine (green), freshwater (light blue), or unknown (white). Using the parsimony tree from Sansom (2009) it is necessary to envisage five changes in palaeoenvironments from marine to freshwater. The deep branches of the tree do not however have strong support (Sansom 2009). A backbone constraint tree for just one freshwater invasion ([*Cephalaspis* + Benneviaspidida + Zenaspidida + *Ilemoraspis* + Kiaeraspididae]) is highly unparsimonious (9 additional character changes required). Far more plausible is monophyly of what could be termed the ‘eucornuates’, [*Cephalaspis* + Benneviaspidida + Zenaspidida ± *Ilemoraspis*] which requires only two or three additional morphological character changes from the most parsminonious solution (depending on inclusion or exclusion of *Ilemoraspis*; alternative phylogenetic solution in green). Furthermore, eucornuate monophyly reduces the number of Wenlock to Devonian ghost ranges to either one or two depending upon whether *Ilemoraspis* is included (orange branches in figure). At least two Devonian invasions of freshwater habitats have therefore occurred on the basis of phylogeny - one in Kiaeraspidoidae and one in what is called here the ‘eucornuates’ (*Cephalaspis*, Zenaspidida, Benneviaspidida and possibly *Ilemoraspis*). There are two exceptions to this pattern - *Camptaspis* and *Ateleaspis*. *Camptaspis* is found in brackish deltaic environments of the Water Canyon Formation (lower Grassy Flat member) of Utah and the Beartooth Butte Formation at Beartooth Butte (Blieck and Cloutier, 2000; Denison, 1952). It has been suggested that the vertebrates of this fauna, including the Osteostraci, may have inhabited fluvial environments and were transported into the hypersaline environments by flooding (Reed, 1997). Such a scenario would be consistent with the fragmentary and worn nature of *Camptaspis* material (pers. obs. RSS). *Camptaspis* therefore needs not represent an inconsistency with the other Devonian freshwater Osteostraci. The Lesmahagow and Hagshaw inliers which yield *Ateleaspis* are confirmed to be terrestrial and freshwater in origin on the basis of both lithology and palynmorphs (Walton and Oliver, 1991; Wellman and Richardson, 1993). *Ateleaspis* is the earliest recorded osteostracan yet the ancestral condition for the group is still resolved as marine/marginal-marine; it is not certain at this time whether *Ateleaspis* represents a true freshwater invasion.


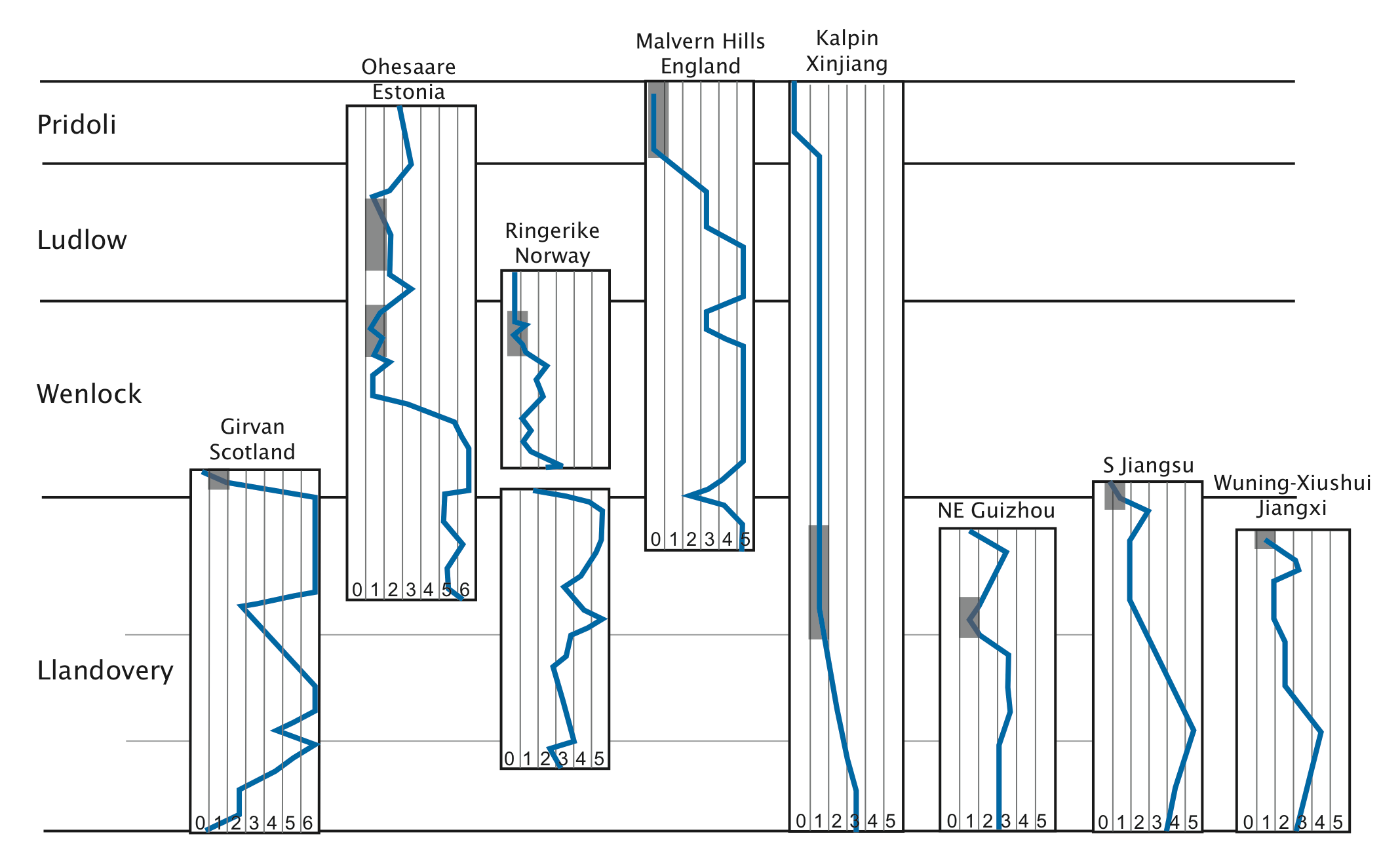
**Supplementary Figure 2.** Sea level curves for Silurian localities yielding Osteostraci and Galeaspida where 0-6 are the benthic assemblage zones (0 is shallow/continental, 6 is deep ocean). Grey shading represents osteotracan and galeaspids yielding horizons. At each locality, the first appearance of ostracoderms is coincident with a change from deeper water environments to shallow water environments. Adapted from Baarli et al (2003), Cocks et al (2003), Rong et al (2003) and Sansom (2008).

| Locality | Formation | Osteostraci | Age | Reference | Environment | Evidence |
| --- | --- | --- | --- | --- | --- | --- |
| Midland Valley and northern, Scotland | Lesmahagow and Hagshaw inliers  *Dictyocaris* band  Arbuthnott group  Caithness Flagstone | *Ateleaspis*  *Hemicyclaspis*  *Cephalaspis*  *Trewinia* | Wenlock, L  Pridoli  Lochkovian,E  Eifelian | Wellman and Richardson, 1993  Westoll, 1945  Lavender and Wellman, 2002; Mykura, 1991  Janvier and Newman, 2005 | Fluvial/lacustrine  Unknown  Fluvial  Lacustrine | Lithology and palynmorph assemblages (Walton and Oliver, 1991; Wellman and Richardson, 1993)  Lithology and palynmorph assemblages (Lavender and Wellman, 2002; Mykura, 1991)  Dineley, 1999 |
| Saaremaa, Gotland and Talsi, East Baltic | Rootsiküla  Paadla  Pernu | Various thyestiids  Various thyestiids  *Balticaspis* | Wenlock, L  Ludlow, E  Eifelian | Blieck and Janvier, 1991  Blieck and Janvier, 1991  Otto and Laurin, 2001 | Shallow marine lagoon  Unknown | Faunal composition (Janvier, 1985a; Märss and Einasto, 1978) |
| Ringerike, Norway | Sundvollen (zone 10)  Holmstrand | *Aceraspis, Hirella and Tyriaspis*  *Hemicyclaspis* | Wenlock, L  Pridoli | Baarli et al., 2003; Davies et al, 2005; Heintz, 1974; Worsley, 1984 | Marginal-marine intertidal  Low energy beach | Lithology and trace fossils (Davies et al., 2006; Davies et al., 2005)  Lithology and trace fossils (Davies et al., 2006) |
| Welshborders, UK | “Downtonian”  “Dittonian” | Some thyestiids, *Hemicyclaspis*  *Various cornuates* | Pridoli  Lochkovian | Allen and Tarlo, 1963  Blieck and Cloutier, 2000 | Brackish delta/  intertidal flats  Fluvial | Lithology and invertebrate fauna (Allen and Tarlo, 1963)  Lithology and Sr isotopes (Allen and Tarlo, 1963; Schmitz et al., 1991) |
| Artic islands,  Canada | Somerset Island  Upper Peel Sound | *Hemicyclaspis*  *Various cornuates* | Pridoli  Lochkovian | Dineley, 1968  Dineley, 1994; Elliot, 1984 | Intertidal mudflat  Fluvial/lacustrine | Miall et al., 1978 |
| Timan Urals,  Russia | Eptarma (*K. timanicus*)  Takarta | *Timanaspis*  *Dobraspis* | Pridoli, U  Emsian, L | Blieck and Janvier, 1991  Rzhonsnitskaya, 1988 | Low sea level  Unknown | Baarli et al., 2003 |
| Tuva and Khakassia,  Russia | Khondergei  Samagaltai  Matarakskaya  Ilemorovskaya | *Tannuaspis*  *Tannuaspis*  *Ilemoraspis*  *Ilemoraspis* | Lochkovian  Lochkovian  Emsian, L  Givetian, U | S. Rodygin, pers. com.  S. Rodygin, pers. com.  Sansom et al., in 2008  Afanasieva and Janvier, 1985 | Unknown  Unknown  Lacustrine  Unknown | Sansom et al. 2008 |
| Spitsbergen, Svalbard | Red Bay group  Wood Bay group | *Various cornuates*  *Various cornuates* | Lochkovian  Pragian and Emsian, L | Blieck and Cloutier, 2000; Blomeier et al., 2003 | High-energy fluvial and broad alluvial plains | Sedimentology (Blomeier et al., 2003; Wisshak et al., 2004) |
| Severnaya Zemlya, Russia | Samoylovich  Ustye Spokoinoi  Spokojnaya | *Tremataspis and Oeselaspis*  *Various cornuates* | Wenlock, U  Ludlow, L  Pragian, L | Afanassieva and Karatjute-Talimaa, 1998  Männik et al., 2002; Mark-Kurik and Janvier, 1995 | Unknown  Terrigenous | Männik et al., 2002 |
| Podolia, Ukraine | Tyver (Old Red I)  Dniester (Old Red III) | *Various cornuates*  *Various cornuates* | Lochkovian,U  Pragian, L | Belles-Isles and Janvier, 1984; Blieck and Cloutier, 2000 | Fluvial/lacustrine  Fluvial/lacustrine | Trace fossils and Sr isotopes (Schmitz et al., 1991; Uchman et al., 2004) |
| Artois, France | Pernes | *Pattenaspis* | Lochkovian | Blieck and Janvier, 1989 | Unknown |  |
| Nova Scotia, Canada | Knoydart | *“Cephalaspis” novasoctiae* | Lochkovian | Blieck and Cloutier, 2000; Denison, 1955 | Unknown |  |
| Rhineland, Germany |  | *Pattenaspis*  *Hildenspis* | Lochkovian,U Emsian, L | Friman and Janvier. 1986 | Unknown |  |
| Gaspé penisula, Canada | Battery Point  La Garde  Escuminac | *Yvonaspis*  *Yvonaspis*  *Escuminaspis and Levesquaspis* | Emsian  Emsian  Frasnian | Belles-Isles, 1989b; Hesse and Sawh, 1992  Cloutier et al., 1996; Janvier and Arsenault, 1996 | Fluvial  Coastal marine/ brackish/fresh water | Blieck and Cloutier, 2000; Griffing et al., 2000  Various conflicting evidence (Cloutier et al., 1996; Hesse and Sawh, 1992; Prichonnet et al., 1996) |
| NW Territories, Canada | Delorme assemblage | *Superciliaspis and Waengsjoeaspis* | Lochkovian | Adrain and Wilson, 1994; Zorn et al., 2005 | Hyposaline lagoon | Lithology and invertebrate fauna (Dineley and Loeffler, 1976; Zorn et al., 2005) |
| Utah/Wyoming, USA | Water Canyon  Beartooth Butte | *Camptaspis* | Emsian | Blieck and Cloutier, 2000; Denison, 1952 | Peritidal delta/estuarine | Blieck and Cloutier, 2000 |

**Supplementary Table 1.** Osteostracan localities with fauna, ages and environments.

**Supplementary References**

Adrain, J.A. and Wilson, M.V.H., 1994. Early Devonian cephalaspids (Vertebrata: Osteostraci: Cornuata) from the Southern Mackenzie Mountains, N.W.T., Canada. *Journal of Vertebrate Paleontology*, 14(3): 301-319.

Afanassieva, O.B. and Janvier, P., 1985. *Tannuaspis, Tuvaspis* and *Ilemoraspis,* endemic osteostracan genera from the Silurian and Devonian of Tuva and Khakassia (USSR). *Geobios*, 18: 493-506.

Afanassieva, O.B. and Karatajute-Talimaa, V.N., 1998. New osteostracans (Agnatha) from the Silurian and Lower Devonian of the Severnaya Zemlya Archipelago (Russia). *Paleontological Journal: Translated from Paleontologicheska Zhurnal* (Moscow), 32(6): 605-610.

Allen, J.R.L. and Tarlo, L.B., 1963. The Downtonian and Dittonian facies of the Welsh Borderland. *Geological Magazine*, 100(2): 129-155.

Baarli, G.B., Johnson, M.E. and Antoshkina, A.I., 2003. Silurian stratigraphy and paleogeography of Baltica. In: E. Landing and M.E. Johnson (Editors), *Silurian Lands and Seas: Paleogeography Outside of Laurentia*. New York State Museum Bulletin, Albany, pp. 3-33.

Belles-Isles, M., 1989b. *Yvonaspis*, nouveau genre d'Osteostraci (Vertebrata, Agnatha) du Dévonien (Emsien-Eifélien) des Grés de Gaspé (Québec, Canada). *Canadian Journal of Earth Sciences*, 26: 2396-2401.

Belles-Isles, M. and Janvier, P., 1984. Nouveaux ostéostracés du Dévonien inférieur de Podolie (RSS d'Ukraine). *Acta Palaeontologica Polonica*, 29(3): 195-208.

Blieck, A.R.M. and Cloutier, R., 2000. Biostratigraphic correlations of Early Devonian vertebrate assemblages of the Old Red Sandstone Continent. In: A.R.M. Blieck and S. Turner (Editors), *Palaeozoic vertebrate biochronology and global marine/non-marine correlation (final report of IGCP 328)*. Courier Forschungsinstitut Senckenberg, Frankfurt am Main, pp. 223-269.

Blieck, A.R.M. and Janvier, P., 1989. Vertébrés agnathes du Dévonieen Inférieur de l'Artois (Pas-de-Calais, France): Implication biostratigraphiques. *Annales de Paléontologie*, 75(3): 125-167.

Blieck, A.R.M. and Janvier, P., 1991. Silurian vertebrates. In: M.G. Bassett, P.D. Lane and D. Edwards (Editors), *The Murchison Symposium.* The Palaeontological Association, London, pp. 345-390.

Blomeier, D., Wisshak, M., Dallman, W., Volohonsky, E. and Freiwald, A., 2003. Facies analysis of the Old Red Sandstone of Spitsbergen (Wood Bay formation): Reconstruction of the depositional environments and implications of basin development. *Facies*, 49(2): 151-174.

Cloutier, R., Loboziak, S., Candilier, A.-M. and Blieck, A.R.M., 1996. Biostratigraphy of the Upper Devonian Escuminac Formation eastern Québec, Canada: a comparative study based on miospores and fishes. *Review of Palaeobotany and Palynology*, 93: 191-215.

Cocks, R.M., McKerrow, W.S. and Verniers, J., 2003. The Silurian of Avalonia. In: E. Landing and M.E. Johnson (Editors), *Silurian lands and seas: Paleogeography outside of Laurentia*. New York State Museum Bulletin, Albany, pp. 35-53.

Davies, N.S., Sansom, I.J. and Turner, P., 2006. Trace fossils and paleoenvironments of a Late Silurian marginal-marine/alluvial system: the Ringerike Group (Lower Old Red Sandstone), Oslo Region, Norway. *Palaios*, 21: 46-62.

Davies, N.S., Turner, P. and Sansom, I.J., 2005. A revised stratigraphy for the Ringerike Group (Upper Silurian, Oslo Region). *Norwegian Journal of Geology*, 85: 193-201.

Denison, R.H., 1952. Early Devonian fishes from Utah. Part I. Osteostraci. *Fieldiana*, *Geology*, 11(6): 265-287.

Denison, R.H., 1955. Early Devonian vertebrates from the Knoydart Formation of Nova Scotia. *Fieldiana, Zoology*, 37: 449-464.

Dineley, D.L., 1968. Osteostraci from Somerset Island. *Bulletin of the Geological Survey of Canada*, 165: 47-63.

Dineley, D.L., 1994. Cephalaspids from the Lower Devonian of Prince of Wales Island, Canada. *Palaeontology*, 37(1): 61-70.

Dineley, D., 1999. Late Silurian fossil fishes sites of the Welsh Borders. In: D. Dineley and S.J. Metcalf (Editors), *Fossil fishes of Great Britain*. Joint Nature Conservation Committee, Peterborough, pp. 63-106.

Dineley, D.L. and Loeffler, E.J., 1976. Ostracoderm faunas of the Delorme and associated Siluro-Devonian formations of North West Territories Canada. *Special Papers in Palaeontology*, 18: 214.

Elliott, D.K., 1984. Siluro-Devonian fish biostratigraphy of the Canadian Arctic Islands. *Proceedings of the Linnean Society of New South Wales*, 107(3): 197-209.

Friman, L. and Janvier, P., 1986. The Osteostraci (Vertebrate, Agnatha) from the Lower Devonian of the Rhenish Slate Mountains, with special reference to their anatomy and phylogenetic position. *Neues Jahrbuch für Geologie und Paläontologie, Abhandlungen*, 173(1): 99-116.

Griffing, D.H., Bridge, J.S. and Hotton, C.L., 2000. Coastal-fluvial palaeoenvironments and plant palaeoecology of the Lower Devonian (Emsian), Gaspé Bay, Québec, Canada. In: P.F. Friend and B.P.J. Williams (Editors), *New Perspectives on the Old Red Sandstone*. Geological Society, London, pp. 61-84.

Heintz, A., 1974. Addtional remarks about *Hemicyclaspis* from Jeløya, southern Norway*. Norsk Geologisk Tidsskrift*, 54: 375-384.

Hesse, R. and Sawh, H., 1992. Geology and sedimentology of the Upper Devonian Escuminac Formation, Quebec, and evaluation of its paleoenvironment: lacustrine versus estuarine turbidite sequence. *Atlantic Geology*, 28: 257-275.

Janvier, P., 1985a. Environmental framework of the diversification of the Osteostraci during the Silurian and Devonian. *Philosophical Transactions of the Royal Society of London. B*, 309: 259-272.

Janvier, P. and Arsenault, M., 1996. Osteostraci. In: H.P. Schultze and R. Cloutier (Editors), *Devonian Fishes and Plants of Miguasha*, Quebec, Canada. Friedrich Pfeil, Munich, pp. 123-133.

Janvier, P. and Newman, M.J., 2005. On *Cephalaspis magnifica* Traquair, 1893, from the Middle Devonian of Scotland, and the relationships of the last osteostracans. *Transactions of the Royal Society of Edinburgh: Earth Sciences*, 95: 511-525.

Lavender, K. and Wellman, C.H., 2002. Lower Devonian spore assemblages from the Arbuthnott Group at Canterland Den in the Midland Valley of Scotland. *Review of Palaeobotany and Palynology,* 118: 157-180.

Männik, P., Menner, V.V., Matukhin, R.G. and Kuršs, V., 2002. Silurian and Devonian strata on the Severnaya Zemlya and Sedov archipelagos (Russia). *Geodiversitas*, 24(1): 99-122.

Mark-Kurik, E. and Janvier, P., 1995. Early Devonian osteostracans from Severnaya Zemlya, Russia. *Journal of Vertebrate Paleontology*, 15(3): 449-462.

Märss, T. and Einasto, R., 1978. Distribution of vertebrates in deposits of various facies in the North Baltic Silurian. *Eesti NSV Teaduste Akadeemia Toimetised. Köide Geoloogia*, 27(1): 16-22. [In Russian with English summary].

Miall, A.D., Kerr, J.W. and Gibling, M.R., 1978. The Somerset Island Formation: an Upper Silurian to ?Lower Devonian intertidal/supratidal sucessian, Boothia uplift region, Arctic Canada. *Canadian Journal of Earth Sciences,* 15: 181-189.

Mykura, W., 1991. Old Red Sandstone. In: G.Y. Craig (Editor), *Geology of Scotland*. The Geological Society, London, pp. 297-346.

Otto, M. and Laurin, M., 1999. Osteostracan tesserae from the Baltic Middle Devonian: morphology and microanatomy. *Neues Jahrbuch für Geologie und Paläontologie. Monatshefte*(8): 464-476.

Prichonnet, G., Di Vergilio, M. and Chidiac, Y., 1996. Stratigraphical, sedimentological and paleontological context of the Escuminac Formation: Paleonenvironmental hypotheses. In: H.P. Schultze and R. Cloutier (Editors), *Devonian Fishes and Plants of Miguasha*, Quebec, Canada. Verlag Dr. Friedrich Pfeil, München.

Reed, R.C., 1997. *Stratigraphy and sedimentology of the Lower and Middle Devonian Water Canyon Formation, northern Utah*. Unpublished Masters Thesis, Northern Arizona University, Flagstaff.

Rong, J.-Y. et al., 2003. Silurian paleogeography of China. In: E. Landing and M.E. Johnson (Editors), *Silurian Lands and Seas: Paleogeography outside of Laurentia*. New York State Museum Bulletin, Albany, pp. 243-297.

Rzhonsnitskaya, M.A., 1988. Biostratigraphic scheme of the Devonian of the Russian platform. In: N.J. McMillan, A.F. Embry and D.J. Glass (Editors), *Devonian of the World (proceedings of the second international symposium on the Devonian system, Calgary, Canada)*. Canadian Society of Petroleum Geologists, Calgary, pp. 691-701.

Sansom, R. S. 2008. The origin and early evolution of the Osteostraci (Vertebrata): A phylogeny for the Thyestiida. *Journal of Systematic Palaeontology,* 6: 217-332.

Sansom, R. S. 2009. Phylogeny, classification and character polarity of the Osteostraci (Vertebrata). *Journal of Systematic Palaeontology,* 7: 95-115.

Sansom, R. S., Rodygin, S. A. and Donoghue, P. C. J. 2008. The anatomy, affinity and phylogenetic signficance of *Ilemoraspis kirskinskayae* (Osteostraci) from the Devonian of Siberia. *Journal of Vertebrate Paleontology,* 28: 613-625.

Schmitz, B., Åberg, G., Werdelin, L., Forey, P.L. and Bendix-Almgreen, S.E., 1991. ^87^Sr/^86^Sr, Na, F, Sr, and La in skeletal fish debris as a measure of the paleosalinity of fossil-fish habitats. *Geological Society of America Bulletin*, 103: 786-794.

Uchman, A., Drygant, D., Paskowski, M., Porębski, S. and Turnau, E., 2004. Early Devonian trace fossils in marine to non-marine redbeds in Podolia, Ukraine: palaeoenvironmental implications. *Palaeogeography, Palaeoclimatology, Palaeoecology*, 214: 67-83.

Walton, E.K. and Oliver, G.J.H., 1991. Lower Palaeozoic- Stratigraphy. In: G.Y. Craig (Editor), *Geology of Scotland*. The Geological Society, London, pp. 161-193.

Wellman, C.H. and Richardson, J.B., 1993. Terrestrial plant microfossils from Silurian inliers of the Midland Valley of Scotland. *Palaeontology*, 36(1): 155-193.

Westoll, T.S., 1945. A new cephalaspid fish from the Downtonian of Scotland, with notes on the structure and classification of ostracoderms. *Transactions of the Royal Society, Edinburgh*, 61(2): 241-357.

Wisshak, M., Volohonsky, E., Seilacher, A. and Freiwald, A., 2004. A trace fossil assemblage from fluvial Old Red deposits (Wood Bay Formation; Lower to Middle Devonian) of NW-Spitsbergen, Svalbard. *Lethaia*, 37: 149-163.

Worsley, D. et al., 1983. The Silurian succession of the Oslo region. *Norges Geologiske Undersøkelse*, 384: 1-57.

Zorn, M.E., Caldwell, M.W. and Wilson, M.V.H., 2005. Lithological analysis of the Lower Devonian vertebrate-bearing beds at the MOTH locality, N.W.T., Canada: insights to taphonomy and depositional setting. *Canadian Journal of Earth Sciences*, 42: 763-775.
